# Open Window: When Easily Identifiable Genomes and Traits Are in the Public Domain

**DOI:** 10.1371/journal.pone.0092060

**Published:** 2014-03-19

**Authors:** Misha Angrist

**Affiliations:** 1 Institute for Genome Sciences and Policy, Duke University, Durham, North Carolina, United States of America; 2 Program in Science and Society, Duke University, Durham, North Carolina, United States of America; 3 Sanford School of Public Policy, Duke University, Durham, North Carolina, United States of America; Vanderbilt University, United States of America

## Abstract

*“One can't be of an enquiring and experimental nature, and still be very sensible.”*

- Charles Fort [1]

As the costs of personal genetic testing “self-quantification” fall, publicly accessible databases housing people's genotypic and phenotypic information are gradually increasing in number and scope. The latest entrant is openSNP, which allows participants to upload their personal genetic/genomic and self-reported phenotypic data. I believe the emergence of such open repositories of human biological data is a natural reflection of inquisitive and digitally literate people's desires to make genomic and phenotypic information more easily available to a community beyond the research establishment. Such unfettered databases hold the promise of contributing mightily to science, science education and medicine. That said, in an age of increasingly widespread governmental and corporate surveillance, we would do well to be mindful that genomic DNA is uniquely identifying. Participants in open biological databases are engaged in a real-time experiment whose outcome is unknown.

## Do You Want to Know a Secret?

If there is an abiding and irrefutable lesson to be drawn from global events of the last couple of years, it might be this (drum roll please): secrets are hard to keep. Perhaps harder than ever. Whether one finds his actions reprehensible or heroic, Edward Snowden managed to get his paws on an unprecedented volume of classified documents detailing the extent of American government-sponsored surveillance efforts and to share those documents far and wide. Even the well-financed and heavily encrypted appropriators of others' secrets could not keep their own activities secret [2].

In a sense each of us carries a singular “classified” document written in each one of our trillions of nucleated cells (with slight but significant variations among people). This document contains many thousands of lengthy words derived from a simple four-letter chemical alphabet along with a whole bunch of gibberish (okay: *functional* gibberish, if you insist [3]). Until recently we labored under the illusion that such information was kept safe from others' (and our own!) prying eyes by arcane and erratically enforced laws [4], [5] together with researchers' presumably unassailable anonymization/de-identification algorithms [6].

But then came the truth-tellers (or leakers, depending upon one's political leanings). Yaniv Ehrlich—biology's answer to Snowden?—and colleagues demonstrated conclusively what most everyone in the field had long realized but relatively few were willing to admit out loud: *DNA sequence is identifying*. And unless you're a monozygotic twin, it is *uniquely* identifying. If I have your genome then not only can I learn some stuff about your traits and ancestry, but if I have the right skills and make a bit of an effort then there is a decent chance I will be able to figure out exactly who you are [7]. Such is the nature of six-billion-character barcodes.

## Responses to Open Genomes

What is the community to do in the face of this revelation (however unsurprising)? One response is to double down on technology: invest in and insist upon better encryption. Take the fence and electrify it. If it is already electrified, increase the voltage. Another approach is to make the consequences for unlawful re-identification more severe. If someone is caught stealing personal data—be it a credit card or a genome—throw the book at him: put meaningful deterrents in place.

A third approach (not at all mutually exclusive with the first two), articulated by Harvard geneticist George Church [8] and instantiated by his Personal Genome Project [9], is to throw up one's hands and simply make one's genotypes and phenotypes public. This response involves saying to patients and research participants in the starkest possible terms, “Secrets, especially genetic ones, *are* hard to keep. If you share your own DNA data online then you are putting yourself (and perhaps your family members) at greater risk (no we don't know how much greater) for discrimination and various other bad things (e.g., discovery of non-paternity). If you're uncomfortable with that, that is absolutely fine—most people are! But in that case then you should probably not be participating in our project, which involves sharing one's own personal data in a public database without much in the way of electrified fences. If, on the other hand, you have gotten this far and are still game to join our band of not-sensible biological exhibitionists putting it all out there for the benefit of science, please sign here.”

This is essentially what Greshake et al. [10] have done. They have created openSNP (opensnp.org), a public not-for-profit online database that allows individuals to upload their own single-nucleotide-polymorphism genotypes (typically from direct-to-consumer genotyping services like 23andMe) and phenotypes that are then accessible to anyone online. One can attach a pseudonym to one's data, but the site rightly cautions would-be participants that such a move is unlikely to afford one much protection from determined bad actors. It also warns potential enrollees about the permanence of data uploaded to the internet; the risk of probabilistic familial disclosures; the potential for discrimination based on genotype; and the possibility of discovering bad news within one's DNA, either now or in the future (https://opensnp.org/disclaimer). In short, it's not for everyone.

OpenSNP is not the first site of its kind. SNPedia, a wiki-based bioinformatics site that houses a database of SNPs and SNP-chip-based reports for individual users (including me), launched in 2007 [11]. But SNPedia does not have a mechanism for aggregating individual-level phenotypic data. The Personal Genome Project posts individual genome, exome, SNP and trait data [9] at the GET-Evidence site (evidence.personalgenomes.org/); however, at present it does not provide an application programming interface, which means data downloads are somewhat cumbersome [Madeleine Price Ball, personal communication, 28 January 2014]. Without large-scale institutional support, it is hardly surprising that ad hoc bottom-up collections of biological data have some Rube Goldberg aspects to them. I should also note that while the authors complain that the Harvard-based PGP is limited to United States citizens [10], there are now four international PGP sites that have received IRB approval and more sites are in the works [Jason Bobe, personal communication, 28 January 2014].

## Man's Search for Meaning

While the value of these sites as indispensable repositories of genomic data constantly enriched via crowdsourcing has yet to be realized fully, openSNP arrives at an especially propitious moment. In November 2013 the United States Food and Drug Administration sent a strongly worded cease-and-desist letter to 23andMe, the leading commercial direct-to-consumer genetics provider, complaining that the company's genetic testing product was an unapproved medical device being deployed inappropriately as a diagnostic [12]. Shortly thereafter, in accordance with the agency's demands, 23andMe stopped providing interpretations of its 254 health-related SNP genotypes to customers [13].

In the media firestorm that followed, what received less attention was FDA's concession that *individuals should have unfettered access to their own raw genomic data* [14], a position that is in accord even with the radical openness espoused by the PGP. As Lunshof et al. pointed out recently, “Access to raw data is independent from the prospective delivery of interpreted information…” [15].

Thus, with its raw-data stance FDA offers an opening for nonprofit crowdsourced personal genomics sites like openSNP, PGP and SNPedia. While openSNP mines the web for genome-wide-association data and related publications just as 23andMe does, it does not make definitive statements about individuals' risks or susceptibilities, nor does it make claims about their broader health or the Awesome Diagnostic, Prognostic and Curative Power of Genetics. It simply aggregates the raw data and makes it available to anyone and everyone for any purpose.

## Read the Fine Print

Does such availability expose participants to risk? Absolutely. So does shopping at Target or revealing intimate details about oneself on social media sites. The University of California-Berkeley's Steve Brenner has warned us that we should be prepared for “the big genome leak.” He concedes, however, that, “The effects might be uncomfortable but would probably reveal less than a typical Google search history.” [16] At the moment he is likely correct. The evidence for genetic discrimination in insurance, for example, is spotty [17], but obviously that could change at any time, especially in places like the United States where there is no single-payer healthcare system.

The take-home message is one I try to impart to my teenage children: if someone asks you for *personal data of any kind*, it is incumbent upon you to be mindful of what it is you are agreeing to. Terms of service and consent forms: pause before clicking through them. If you do not understand them, ask questions. I find sites like Terms of Service; Didn't Read (http://tosdr.org/) to be an extremely helpful reminder of bargains that can sometimes feel both liberating and Faustian.

## Hypotheses and Conversations

What about the usefulness of all this openness? In the case of GET-Evidence, the availability of whole genomes has already turned up clinically relevant (and admittedly unwelcome but potentially actionable) variants [18]. But what about lower-resolution SNP scans: while their clinical value to individuals is suspect, can freely accessible SNP-chip data still lead to dramatic medical breakthroughs? Given the relative paucity of GWAS results that have made their way to the clinic thus far [19], I reckon a large dose of skepticism is in order. But no hypothesis was ever disproved in the absence of data.

In any case, I think it would behoove us to do more than just swing for the translational fences. Until consumer SNP chips include exhaustive panels of variants that cause single-gene disorders, I suspect the real value of sites like openSNP will be as teaching tools and focal points for discussion: what might we do with an expanding reservoir of unfettered self-selected genomic data and self-reported phenotypes? What can current human-participant regulatory regimes learn from these collections? What happens when we declassify our own biological documents en masse? Enquiring minds want to know.
